# Kreon^®^ (Creon^®^) vs. Lipancrea^®^: *In Vitro* Comparison of Two Encapsulated Pancreatin Preparations

**DOI:** 10.3390/ph15121570

**Published:** 2022-12-16

**Authors:** Sven Hartmann, Grazyna Rydzewska, J. Enrique Domínguez-Muñoz

**Affiliations:** 1 Viatris Healthcare GmbH, Freundallee 9A, D-30173 Hannover, Germany; 2 Inflammatory Bowel Disease Unit, Clinical Department of Internal Medicine and Gastroenterology, Central Clinical Hospital of the Ministry of Internal Affairs and Administration, 02-507 Warsaw, Poland; 3 Department of Gastroenterology and Hepatology, University Hospital of Santiago de Compostela, 15706 Santiago de Compostela, Spain

**Keywords:** pancreatic enzyme supplement, exocrine pancreatic insufficiency, enzyme activity, pharmacokinetics, *in vitro*, enzyme release, interchangeability

## Abstract

Kreon^®^ (Creon^®^) and Lipancrea^®^ are pancreatic enzyme supplements indicated in the treatment of exocrine pancreatic insufficiency. In order to determine their interchangeability, an *in vitro* comparison of their physical properties and enzymatic activity was carried out. Capsule fill weight and particle size were also determined in order to establish their physical properties. Amylase, lipase and protease activities, lipase release at different pHs and the dissolution time of pellets were assessed for enzymatic analysis. The length range of Kreon^®^ and Lipancrea^®^ pellets was 1.1–2.2 mm and 1.5–2.8 mm, respectively. Protease activity was below the label claim for Lipancrea^®^ and above for Kreon^®^ presentations. Lipase and amylase activity were equal to or higher than the label claim in both preparations. In dissolution experiments simulating the stomach passage, significant release of lipase activity was observed for Lipancrea^®^ (% actual activity: 41% for Lipancrea^®^ 8000; 21% for Lipancrea^®^ 16000) after 60 min at pH 5.0. No release of lipase activity was observed for Kreon^®^ at that particular pH. Enzyme release for Lipancrea^®^ at pH 6.0 was generally slower than for Kreon^®^ and seemed to be influenced by the preceding incubation at lower pH. More than 85% of Kreon^®^ and Lipancrea^®^ dissolved in a pH 6.0 phosphate buffer within 20 min. Despite the similarities of the enzyme content on the respective labels, Kreon^®^ and Lipancrea^®^ differ in pellet size, enzymatic activity and release. This may impact their therapeutic efficacy and, therefore, may limit their interchangeability.

## 1. Introduction

Exocrine pancreatic insufficiency (EPI) is a common consequence of pancreatic diseases (e.g., chronic pancreatitis, cystic fibrosis, acute necrotizing pancreatitis, pancreatic cancer) and gastric and pancreatic surgical resection [1,2]. EPI is characterized by inadequate synthesis, secretion and/or intraluminal activity of pancreatic enzymes, resulting in maldigestion that leads to a deterioration of the patient’s nutritional status [3]. This, in turn, is associated with an increased risk of infection, cardiovascular disease, sarcopenia and osteoporosis, and can lead to increased morbidity, reduced patient quality of life (QoL), and increased mortality [1,2,4,5]. Patients with EPI with nutritional deficiencies, those who lose weight, and those with maldigestion-related symptoms are classically and generally considered to require pancreatic enzyme substitution therapy [1]. Dosages to treat EPI are selected individually and depend on multiple variable factors. Some of them, including body mass and severity of the condition, can be considered stable—they change only moderately over time. There are, however, additional, highly variable factors like amount and composition of the meal, which also have to be considered by the patient and they affect the dose that is administered. Consequentially, patients are normally not on a fixed-dose regimen but rather tailor the therapy to their current situation.

Löhr et al. [6] defined the requirements that an ideal pancreatic enzyme supplement for oral therapy should meet based on the physiological digestive process. These requirements included: pleasant taste and odor-masking of the pancreatic extract, optimal mixing with the chyme in the stomach, resistance to gastric juices, particle size less than 1.7 mm in order to pass the pylorus, rapid enzyme release in the small intestine at approximately pH 6.0, large specific surface area, conformity of ingredients with label declaration, and high batch-to-batch consistency. Löhr et al. pointed out the importance of knowing to what extent the different pancreatin preparations differ with regard to their properties and efficacy in order to determine their interchangeability. They analyzed 13 pancreatin presentations available in Europe and concluded that, despite the fact that all enzyme preparations showed partially high lipase activities relative to the declared dose and similar degrees of resistance to simulated gastric acid, there were remarkable differences with respect to particle size distribution, specific surface area, and release kinetics, which could affect their efficacy and, therefore, their interchangeability.

Lipancrea^®^ is a pancreatic enzyme supplement commercialized in Poland by Polfa Warsaw S.A. (Warszawa, Poland) with two different presentations available: Lipancrea^®^ 8000 and Lipancrea^®^ 16000. At present, Lipancrea^®^ has limited published clinical data [7,8] and has not previously been compared *in vitro* with other pancreatic enzyme supplements. For this reason, in order to determine its physical properties, enzyme content, enzyme activities, and its compliance with the label claim, Lipancrea^®^ was compared against the globally commercialized Kreon^®^ (Mylan Healthcare. Warszawa, Poland, part of Viatris group, Canonsburg, PA, USA). Besides enzyme content, the galenical formulation is crucial for the efficacy of pancreatin-containing preparations; consequently, the kinetics of enzyme release and dissolution of both preparations were also investigated in this study.

## 2. Results

### 2.1. Physical Properties

#### 2.1.1. Capsule Fill Weight

The mean content weight of 10 capsules was determined for Kreon^®^ and Lipancrea^®^. The results are shown in Table 1.

#### 2.1.2. Particle Imaging and Size Determination

**Figure 1:**
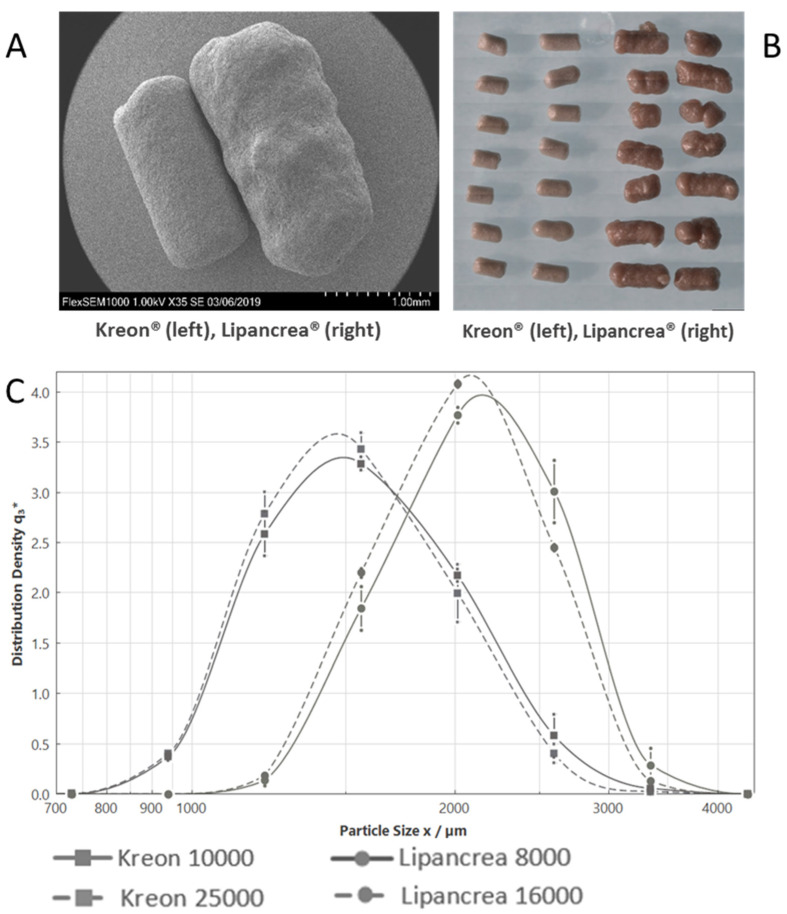
Kreon^®^ and Lipancrea^®^ particle size: (**A**) Kreon^®^ (left) and Lipancrea^®^ (right) scanning microscope image; (**B**) Kreon^®^ (left) and Lipancrea^®^ (right) light microscope image; (**C**) particle size analysis.

The width of both Kreon^®^ drug products was about 0.8 mm, with lengths ranging from 1.1 mm to 2.2 mm (maximum intersect as determined by FERET-Max D [v, 0.1] and D [v, 0.9], respectively). Lipancrea^®^ 16000 pellets were not as uniform, rougher and with larger dimensions from about 1.5 mm up to 2.8 mm (FERET-Max D [v, 0.1] and D [v, 0.9] respectively) (Figure 1, Table 2).

### 2.2. Enzymatic Analysis

#### 2.2.1. Enzyme Activities

The lipase activity measured was equal to or slightly higher than the label claim, while amylase activity was much higher than the label claim for Kreon^®^ and Lipancrea^®^ products. The protease activity measured for Kreon^®^ 10000 and Kreon^®^ 25000 was far above their label claim. Total protease activity measured for Lipancrea^®^ 8000 and Lipancrea^®^ 16000, however, was slightly below their label claim (Table 3).

#### 2.2.2. Enzyme Release

Minimal or no release of lipase activity was observed for Kreon^®^ and Lipancrea^®^ products after 60 min at pH 1.0 and pH 4.0. Significant release of lipase activity, however, was observed for Lipancrea^®^ 8000 and Lipancrea^®^ 16000 after 60 min at pH 5.0, whilst no release of lipase activity was observed for Kreon^®^ 10000 and Kreon^®^ 25000 (Table 4).

**Figure 2:**
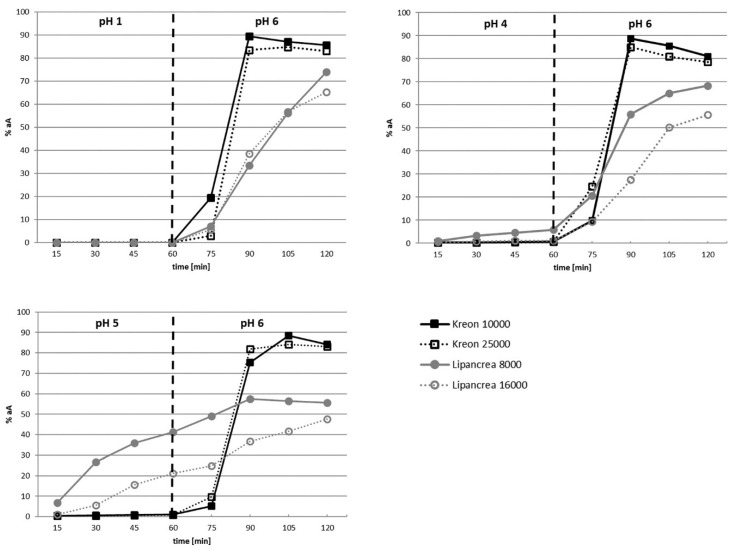
Kinetics of enzyme release at pH 1.0, 4.0, 5.0 and 6.0. NOTE: on the *y*-axis, the enzyme amount is normalized to the actual assay of the respective product to compensate for different strengths (as per label claim) but also for overages up to 10%, which have been observed in the assay (compared with Table 2). The vertical dotted line represents the change in pH.

Upon changing the pH to 6.0, immediate enzyme release was observed for Kreon^®^ 10000 and Kreon^®^ 25000, and maximum enzyme release (89% and 85%, respectively) was generally achieved within 30 min (Figure 2).

**Figure 3:**
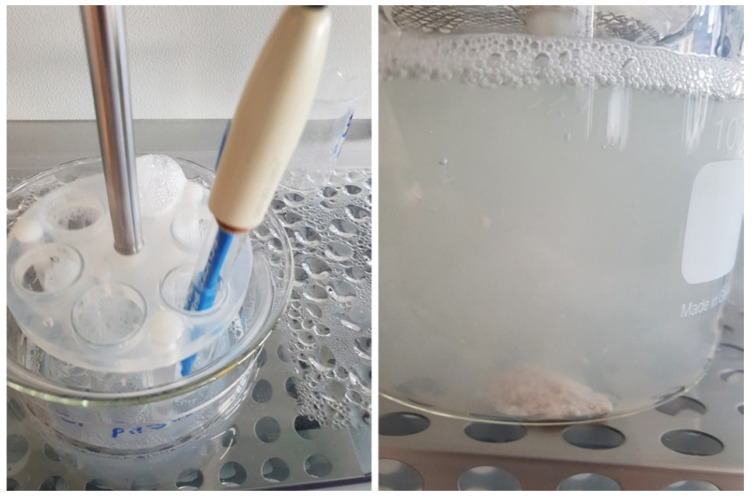
Pictures of Lipancrea^®^ 16000 after 60 min in buffer at pH 6.0. Foaming was observed during the test. After 60 min at pH 6.0, a single sticky mass was present at the bottom of the reaction beaker for Lipancrea^®^.

Enzyme release for Lipancrea^®^ at pH 6.0 was generally slower than for Kreon^®^ and seemed to be influenced by prior incubation at lower pH. Accordingly, when Lipancrea^®^ presentations were first incubated at pH 1.0, maximum enzyme release was not obtained after 60 min at pH 6.0 (74% and 65% for Lipancrea^®^ 8000 and 16000, respectively). When the pH of the initial incubation mixture increased, a decrease in the maximum enzyme release for Lipancrea^®^ at pH 6.0 was observed (Figure 2). It should be noted that after 60 min at pH 6.0, a single sticky mass was present at the bottom of the reaction beaker for Lipancrea^®^ but not for Kreon^®^. In addition, significant foaming was observed for Lipancrea^®^ in phosphate buffer at pH 6.0 but not for Kreon^®^ (Figure 3).

#### 2.2.3. Dissolution Test

**Figure 4:**
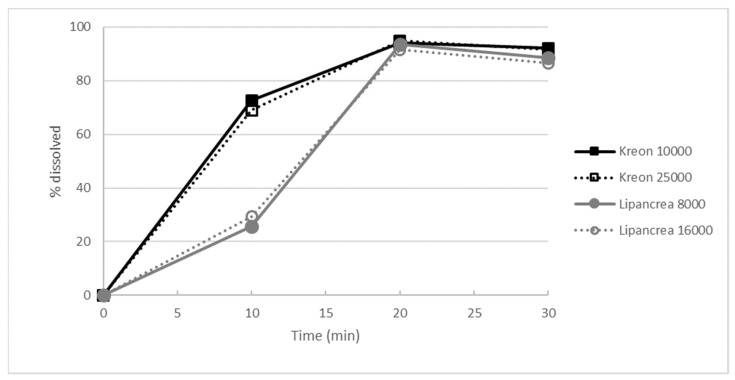
Dissolution profile in phosphate buffer at pH 6.0, as evaluated by the percentage of lipase activity released.

After an initial incubation in simulated gastric fluid for 2 h, the dissolution of Lipancrea^®^ products was significantly slower than that observed for Kreon^®^ products in the initial stages (10 min) at pH 6.0 (*p* < 0.05). Nonetheless, more than 85% of Kreon^®^ and Lipancrea^®^ dissolved in phosphate buffer at pH 6.0 within 20 min, showing that, in principle, all products are able to release their respective enzymes (Figure 4).

## 3. Discussion

This *in vitro* study compared two pancreatic enzyme supplements (Kreon^®^ and Lipancrea^®^), assessing their physical properties, enzyme content, enzyme activities and compliance with the label claim in order to determine their interchangeability. As patients need to adjust the dosage depending on the specific situation and factors like body weight, the severity of PEI, and the type and amount of food, several different product strengths may be combined to reach the desired individual dose.

As the products compared in this study differ in their label strengths, data has been presented relative to label claim to enable an unbiased comparison.

As published by Löhr et al., an ideal pancreatic enzyme supplement should meet several requirements in accordance with the physiological digestive process. One of the key requirements is pellet size, as particles larger than 1.7 mm cannot pass through the pylorus [6]. This is supported by *in vivo* comparisons between different particle size preparations that confirm a greater treatment efficacy with smaller particle sizes (1.0–1.2 mm vs. 1.8–2.0 mm) [9]. In fact, particles larger than 2.0 mm are probably not emptied from the stomach during fed gastric motility together with the chyme [10,11] and are retained for more than 2 h [12]; therefore, smaller particles have a more rapid onset of action and are more effective [13]. Meyer et al. reported that particles should not exceed 1.4 ± 0.3 mm to mimic physiology [14]. Our study has shown differences between Kreon^®^ (10000 and 25000) and Lipancrea^®^ 16000 in terms of particle size, revealing a shorter Kreon^®^ length, and suggesting its easier passage through the pylorus. The cylindrical shape of the particles poses some challenges if their size is to be described in one number. The FERET-Max parameter, as selected here, tends to overestimate particle size slightly but would not compromise the fact that Kreon^®^ particles are smaller than Lipancrea^®^ ones. Due to the bell-shaped curves, the majority of Kreon^®^ particles would meet the 1.7 mm cutoff (Figure 1C; FERET-Max D [v, 0.5]/µm is below 1.7 mm for Kreon^®^ (1566 and 1531 µm for Kreon^®^ 10000 and Kreon^®^ 25000, respectively) but above for Lipancrea^®^ (2120 and 2043 µm for Lipancrea^®^ 8000 and Lipancrea^®^ 16000, respectively)), as defined by Löhr (2009) [6]. The results presented here match data measured previously on Kreon^®^ with a similar method and are in line with measurements obtained using another approach [6,15]. Lipancrea^®^ particle size has not been measured previously.

The conformity of ingredients with the label declaration is another requirement that an ideal pancreatic enzyme supplement should meet [6]. Differences between Kreon^®^ and Lipancrea^®^ were observed in the protease activity measured. Protease activity was slightly below the label claim for Lipancrea^®^ presentations and far above the label claim for Kreon^®^ presentations. In contrast, lipase activity and amylase activity were equal or higher than the label claim in both preparations. This is in line with data published previously on Kreon^®^ [6,15]. It has also been reported previously that certain products in this field do not meet the label claim for lipase [6,15] or amylase [6].

The physiochemical properties of the coating of the preparations are among the most critical determinants of the efficacy of enzyme substitution therapies [16]. The preparations need to be enteric-coated, as pancreatic lipase is not stable in an acidic medium and would degrade. When pancreatic enzymes are ingested simultaneously with food, the pellets should mix with gastric chyme without releasing the enzymes at an acidic pH. When the pH increases to approximately 6.0, usually in the duodenum, the pellets are designed to release the enzymes [6,16]. In addition to the number of active enzymes present, an immediate release of the enzymes within the proximal gut is necessary in order to achieve a therapeutic effect. The entero-coated formulation has the function of protecting the enzymes contained in the pellets during the gastric passage. The pH in gastric passage ranges from 1.0 to 2.0; however, this range can reach higher values due to the buffering capacity of ingested food. Thus, a temporary increase of the pH to 5.0 is not unusual [17,18], resulting in the disintegration of the coating and, therefore, the release of enzymes. Thus, in order to prevent the deactivation of pancreatic enzymes within the stomach, the enzymes should not be released within a pH range of 1.0 to 5.0. Once the duodenum is reached, with a pH value beyond 6.0, the coating of the particles has to ensure the immediate release of enzymes in order to achieve maximum digestive potency. Differences were observed in the kinetics of enzyme release of our study preparations. While minimal or no release of lipase activity was observed at pH 1.0 and 4.0, a significant release was observed for Lipancrea^®^ at pH 5.0 but not for Kreon^®^. Enzyme release at pH 5.0 can lead to enzyme degradation once gastric pH becomes more acidic again and, thus, to a reduction of the efficacy of the enzyme substitution. In addition, while immediate enzyme release was observed for Kreon^®^ at pH 6.0, Lipancrea^®^ enzyme release seemed to be influenced by the prior pH. More specifically, a decreasing trend in maximum enzyme release was observed when the prior pH increased, showing once again differences between the two products. Furthermore, as mentioned earlier, the entero-coated formulation has the function of protecting the enzymes. This coating is different in both products [19,20,21,22], which could be the root cause for their different behavior during enzyme-release testing. At the same time, observed differences do not originate from the capsule shell, which is a plain hard gelatin capsule with some iron oxides which work as colorants.

Enzyme digestion products are mainly absorbed in the duodenum and jejunum [23]. It normally takes around 60 min for the food mass to reach the jejunum [24]: the more proximal the release, the greater the length of the intestine over which the enzymes are available for digestion and further absorption of their products [25]. Our study shows that Lipancrea^®^ and Kreon^®^ release enzymes significantly differently. Both Kreon^®^ products (10000 and 25000) release a higher level of enzymes in a shorter period, reaching maximum enzyme release (89% and 85% respectively) within 30 min, while Lipancrea^®^ does not reach the level of Kreon^®^, which highlights the large variability in their behavior. Moreover, during the 60 min test interval for enzyme release, the Lipancrea^®^ formulations did not reach peak release, making it difficult to estimate at which gastrointestinal compartment the enzymes would be released and what the delayed enzyme availability would mean for the digestion in patients.

Even though the dissolution of both Lipancrea^®^ products was significantly slower than the one observed for Kreon^®^ at the initial stages (10 min), within 20 min at pH 6.0, more than 85% of all four tested enzyme preparations were dissolved, suggesting that the enzymes are, in principle, released from all formulations but with significantly different kinetics.

Previous studies have also shown that pancreatic enzyme products vary in terms of enzyme content and *in vitro* response to simulated gastric and duodenal conditions. The comparison of 13 European-branded pancreatin presentations found remarkable differences with respect to particle size distribution, specific surface area and release kinetics [6]. What is more, a comparison between different lots of two branded pancreatin presentations [16] or branded and generic preparations [26] suggested that not all pancreatic enzyme replacements are equal, even among pancreatic enzyme formulations with pharmaceutically equivalent labels. In line with these results, Aloulou et al. [25] concluded that pH-sensitive enteric-coated pancreatin products containing similar amounts of enzymes might not be bioequivalent.

## 4. Materials and Methods

### 4.1. Physical Properties

In order to establish the physical properties of pancreatin presentations, capsule fill weight and particle size were determined. The pancreatic enzyme preparations acquired and investigated are shown in Table S1.

#### 4.1.1. Capsule Fill Weight

Ten capsules were individually weighed, preserving their identity. The contents of each capsule were removed, and the empty shells were individually weighed. The net mass of the contents was calculated by subtracting the mass of the shell from the respective gross mass.

#### 4.1.2. Particle Imaging

Particle images were taken using a light microscope and scanning electron microscope (SEM). At the light microscope, samples were mounted on glass microscope slides using clear double-sided adhesive tape and then analyzed using a Zeiss Axio Imager Z2M microscope (Zeiss, Oberkochen, Germany). At the SEM (Hitachi FlexSEM 1000, Hitachi, Tokyo, Japan), samples were mounted on sample stubs (G301P, Agar Scientific, Stansted, UK) using 12 mm carbon tabs (G3347N, 170301, Agar Scientific). The mounted samples were gold coated (5 nm layer) using a Quarom sputter coater (Quorum Technologies, Laughton, UK). Depending on the sample, accelerating voltages between 5 and 15 kV were used to achieve optimal surface detail. Auto-focus and auto-contrast were performed prior to the collection of each image, with manual optimization wherever required. Images were collected at various magnifications.

#### 4.1.3. Particle Size Determination

Analysis was performed using a Sympatec QICPIC (Dynamic Image Analyzer, Sympatec, Clausthal-Zellerfeld, Germany) with a GRADIS Disperser and M8 lens. FERET-Max was selected for the evaluation mode as the maximum particle dimension most relevant for passing through the pylorus. Three replicates with 2 g of capsule contents per replicate were recorded for each batch.

### 4.2. Enzymatic Analysis

In order to perform the enzymatic analysis, the activity of amylase, lipase and protease was assessed. In addition, lipase release at different pH levels and the time of dissolution of the pellets was determined. All methods used have been run according to the European Pharmacopoeia (Ph. Eur.), which lays down binding standards for the testing of pharmaceutical products. Specifically, the experimental setup used here is described in Monograph 0350 on Pancreas Powder [27]. The pancreatic enzyme preparations acquired and investigated are shown in Table S1.

#### 4.2.1. Enzyme Activities

Amylase activity, lipase activity and the total proteolytic activity were analyzed. Two runs with the content of 20 capsules removed from the capsule shell per run were performed.

Starch is hydrolyzed by amylase at pH 6.8 and at a constant temperature (25.0 +/− 0.1 °C) in the presence of sodium chloride (Merck, Darmstadt, Germany). Following this principle, amylase activity was determined by comparing the rate at which a suspension of pancreas powder hydrolyzes a starch solution substrate with the rate at which a suspension of pancreas reference powder (amylase reference standard, Abbott, Chicago, IL, USA) hydrolyzes the same substrate under the same conditions.

Lipase hydrolyzes the triglycerides of olive oil. Lipase activity was determined by comparison of the rate at which a suspension of pancreas powder hydrolyzes a substrate of olive oil emulsion with the rate at which a suspension of the lipase reference standard (Abbott, Chicago, IL, USA) hydrolyzes the same substrate under the same conditions.

Casein is hydrolyzed by protease at pH 7.5 and at 35 °C. The total proteolytic activity of pancreas powder was determined by evaluation of the quantity of non-precipitable peptides by a 5% (m/V) solution of trichloroacetic acid (Merck, Darmstadt, Germany) release per minute from a substrate of casein solution, with the protease reference standard (Abbott, Chicago, IL, USA) used as a reference.

#### 4.2.2. Enzyme Release

In order to prevent the inactivation of pancreatic enzymes within the stomach, no enzymes should be released within a pH range of 1.0 to 5.0. Once pH 6.0 is reached, usually at the duodenum, the coating of the particles has to ensure the immediate release of enzymes in order to reach maximum digestive potency. In order to determine the stability and activity of Kreon^®^ and Lipancrea^®^ pellets, they were removed from the capsule shell. This capsule shell does not contribute to the targeted release properties of the formulation, which is mediated by the coating of the individual pellets. Two samples were agitated in a disintegration apparatus (ZT 304, Erweka, Langen, Germany) in solutions of pH 1.0, pH 4.0 and pH 5.0, respectively, for 60 min at 37 °C. The samples were placed afterward in a disintegration tester in a pH 6.0 solution for 60 min and agitated at 37 °C. Four ml test samples were taken for 2 h every 15 min and stored on ice until analysis. Residual lipase activity was then determined. The results at each time point are presented as a percentage of the actual lipase activity.

#### 4.2.3. Dissolution Test

In order to assess the time to dissolution, pellets were removed from the capsule shell. First, they were exposed to simulated gastric fluid in a rotating basket for 2 h. Then, the pellets were transferred to a phosphate buffer (Merck, Darmstadt, Germany) at pH 6.0 and stirred using a paddle apparatus (100 rpm) for 30 min. Samples were taken at 10, 20 and 30 min, and lipase activity was determined. Two runs were performed, and six capsules were measured individually per run.

### 4.3. Statistical Analysis

Mean and standard deviation (SD) were calculated when physical properties and enzymatic analyses of more than one capsule and/or more than one run were performed. When only two results were available (e.g., enzyme release conducted for two operators), SD was not calculated, and both results are shown. The Student’s *t*-test was used to calculate significant differences (*p* < 0.05 was considered statistically significant).

## 5. Conclusions

The results of this investigation demonstrated that Kreon^®^ and Lipancrea^®^ show differences in particle size, conformity of protease with label declaration, enzyme release at pH 5.0, and percentage of enzyme release at pH 6.0, which calls into question the interchangeability of both presentations.

Current evidence comparing pancreatic preparations leads us to conclude that, in spite of exhibiting the same labeled enzyme content, some preparations may not have the same ability to release active enzymes at the right time and at the right place, i.e., at the duodenum and together with the chyme. Therefore, the interchangeability of these drugs may impact the efficacy of pancreatic enzyme replacement therapy.

The undesired effects resulting from limited interchangeability may be particularly relevant in an area such as EPI treatment, where it is not possible to establish a single fixed-dose, but, on the contrary, patients have to adjust their dose for each meal. Multiple additional variables arising from differences between PERT products additionally increase the complexity of the treatment.

## Figures and Tables

**Table 1:** Capsule fill weight (*n* = 10).

Product	Capsule Fill Weight (mg)	Relative Standard Deviation (%)
Kreon^®^ 10000	252.1	2.7
Kreon^®^ 25000	481.7	1.6
Lipancrea^®^ 8000	139.1	3.9
Lipancrea^®^ 16000	272.3	2.5

**Table 2:** Kreon^®^ and Lipancrea^®^ particle size.

Product	D [v, 0.1]/µm	D [v, 0.5]/µm	D [v, 0.9]/µm
Kreon^®^ 10000	1131	1566	2225
Kreon^®^ 25000	1122	1531	2161
Lipancrea^®^ 8000	1542	2120	2814
Lipancrea^®^ 16000	1506	2043	2740

**Table 3:** Comparison of lipase, amylase and protease activity to label claim.

Enzyme	Product	Label Claim (U)	Activity Measured (U); Mean (SD)	% Label Claim Found; Mean (SD)
Lipase	Kreon^®^ 10000	10,000	10,779 (189)	108 (2)
	Kreon^®^ 25000	25,000	26,025 (463)	104 (2)
	Lipancrea^®^ 8000	8000	8255 (49)	103 (1)
	Lipancrea^®^ 16000	16,000	17,617 (324)	110 (2)
Amylase	Kreon^®^ 10000	8000	12,602 (39)	158 (0)
	Kreon^®^ 25000	18,000	25,063 (194)	139 (1)
	Lipancrea^®^ 8000	5750	7530 (66)	131 (1)
	Lipancrea^®^ 16000	11,500	16,417 (147)	143 (1)
Protease	Kreon^®^ 10000	600	780 (10)	130 (2)
	Kreon^®^ 25000	1000	1480 (22)	148 (2)
	Lipancrea^®^ 8000	450	422 (21)	94 (5)
	Lipancrea^®^ 16000	900	844 (21)	94 (2)

**Table 4:** Comparison of percentage of actual activity released after 60 min at pH 1.0, 4.0 and 5.0.

Product	Mean % Actual Activity Released after 60 min (Individual Values Given in Brackets)		
	pH 1.0	pH 4.0	pH 5.0
Kreon^®^ 10000	0 (0; 0)	1 (0.5; 0.9)	1 (0.8; 1.4)
Kreon^®^ 25000	0 (0; 0)	1 (0.3; 0.8)	1 (0.7; 0.9)
Lipancrea^®^ 8000	0 (0; 0)	6 (7.0; 4.8)	41 (44.6; 37.9)
Lipancrea^®^ 16000	0 (0; 0)	1 (1.0; 1.3)	21 (25.4; 16.8)

## Data Availability

Data is contained within the article or Supplementary Material.

## Supplementary Materials

The following are available online at https://www.mdpi.com/article/10.3390/ph15121570/s1, Table S1: Pancreatic enzyme preparations investigated.
