# Supplementary material for: Kreon® (Creon®) vs. Lipancrea®: In Vitro Comparison of Two Encapsulated Pancreatin Preparations
Source: Pharmaceuticals (Basel). 2022 Dec 16;15(12):1570. doi: 10.3390/ph15121570 (PMC9784799; doi:10.3390/ph15121570)
Supplement: Supplementary file 1 [file pharmaceuticals-15-01570-s001.zip › pharmaceuticals-2045630-supplementary.pdf]

pharmaceuticals

MDPI

Table S 1: Pancreatic enzyme preparations investigated

DRUG PRODUCT	BATCH NO.	TEST RECORDS
**Kreon® 10000 (Mylan Healthcare, Viatris Group)**	57797	Microscopy, particle sizing
	55399	Capsule fill weight<br>Enzymatic analysis
**Kreon® 25000 (Mylan Healthcare, Viatris Group)**	58259	Microscopy, particle sizing
**Lipancrea® 8000 (Polfa Warszawa S.A.)**	55433	Capsule fill weight<br>Enzymatic analysis
	011119	Microscopy, particle sizing
**Lipancrea® 16000 (Polfa Warszawa S.A.)**	02NG0217	Capsule fill weight<br>Enzymatic analysis
	01MP0619	Microscopy, particle sizing
